# *Babesia* and Human Babesiosis

**DOI:** 10.3390/pathogens11040399

**Published:** 2022-03-25

**Authors:** Estrella Montero, Jeremy Gray, Cheryl Ann Lobo, Luis Miguel González

**Affiliations:** 1 Parasitology Reference and Research Laboratory, Centro Nacional de Microbiología, Instituto de Salud Carlos III, Majadahonda, 28220 Madrid, Spain; lmgonzal@isciii.es; 2 UCD School of Biology and Environmental Science, University College Dublin, D04 N2E5 Dublin, Ireland; jeremy.gray@ucd.ie; 3 Department of Blood Borne Parasites, Lindsley F. Kimball Research Institute, New York Blood Center, New York, NY 100065, USA; clobo@nybc.org

*Babesia* is a genus of intraerythrocytic protozoan parasites belonging to the exclusively parasitic phylum Apicomplexa. There are more than a hundred known species of this genus, occurring mainly in mammals but also in birds, and all transmitted by ticks, which are blood-sucking arthropods related to spiders. Ixodid (hard-bodied) ticks are vectors of the vast majority of *Babesia *spp., but a small number are transmitted by argasid (soft-bodied) ticks. For many years, *Babesia *spp. were only known as important parasites of domestic animals and were the first pathogens shown to be transmitted by an arthropod vector when, in 1893, Smith and Kilborne reported the vector role of cattle ticks in redwater fever (babesiosis) in the USA [1]. Human babesiosis was first described in 1957 when it occurred as a fulminant and ultimately fatal infection in a Croatian farmer [2]. More human cases followed over the next 50 years, and at least 4 taxonomically classified *Babesia *species (*B. divergens, B. duncani, B. microti*, and *B. venatorum*) have now been confirmed as zoonotic pathogens, with some others that have not yet been identified to species.

The main pathological event of infection with these parasites is the destruction of erythrocytes, resulting in haemolytic anaemia with added complications due to the release of toxins and waste products into the bloodstream. Further damage to the host can be caused by cytokine storms as the host’s immune system responds to infection. In many respects, the pathology of babesiosis is similar to that of the much better-known disease, malaria, caused by *Plasmodium *spp.

This Special Issue consists of 11 reviews that between them address the global babesiosis situation, babesiosis in Europe, the history and current status of *Babesia microti* in the USA, the disease in relation to sickle cell anaemia, the utility of experimental infections of ticks, transfusion transmission, the significance of major surface antigens, advances in the diagnosis of babesiosis, historical and current approaches to treatment and management, and babesiosis in relation to climate change. Additionally, five research articles are presented addressing the discovery of a new zoonotic genotype of *B. divergens*, the characterisation and function of certain proteins involved in parasite–erythrocyte interaction, the identification of proteases as possible drug targets, and the identity of piroplasms in ticks removed from deer in Portugal.

In their review on the worldwide occurrence of human babesiosis, Kumar et al. [3] draw attention to the fact that this is an emerging zoonosis, with increasing reports of infections caused by the known zoonotic species in new areas, for example, in China, in addition to reporting cases that involve *Babesia *parasites of undetermined species. They conclude that the true number of affected patients is considerably underestimated, particularly in regions where clinical and diagnostic overlap with malaria occurs, and call for improved surveillance and continued research on treatment and prevention. The authors mention climate change as a possible factor in the gradual spread of *B. microti *in the USA, and the role of climate in the epidemiology of zoonotic babesiosis in general is discussed more fully by Gray and Ogden [4], with particular reference to climate effects on the vector ticks. While extensive data suggest that global warming is affecting the distribution of the *Ixodes *vectors, no changes in the current occurrence of zoonotic babesiosis can, as yet, be convincingly attributed directly to climate change, though models suggest that this is only a matter of time.

Hildebrandt et al. [5] discuss European babesiosis in more detail. Compared with the USA, the disease is relatively rare in Europe, but the authors point out that most cases present as medical emergencies, mainly in immunocompromised patients, and particularly in those that are asplenic. Unusually, an attempt has been made to present data on every recorded case that has occurred in the last two decades, with the hope of shedding new light on both the epidemiology of the disease as well as on diagnosis and management. Most human babesiosis cases in Europe are due to *B.* *divergens* and *B.* *microti*, although the true prevalence of the latter is unknown because of the apparent low pathogenicity of European strains of this parasite. There is also uncertainty about the epidemiology of the genuinely pathogenic *B. divergens*, particularly the possible role of red deer as reservoir hosts. This topic is again addressed in a research paper by Fernandez et al. [6], who describe a study in which ixodid ticks removed from deer in a Portuguese nature reserve were analysed for piroplasm infections.

*B. divergens* sequences were detected that were apparently identical to those associated with human and bovine babesiosis, and it is concluded that the most likely source of these parasites was the deer. Other interesting findings include the association of *Theileria *spp. with *Ixodes ricinus* and the occurrence of an exophilic form of the brown dog tick, *Rhipicephalus sanguineus.*

*Babesia microti,* the causal agent in the vast majority of cases, particularly in the USA, is the subject of two reviews. Telford et al. [7] describe in detail the emergence of this pathogen 50 years ago. This is probably the first time that all the salient facts behind the appearance of this pathogen and the subsequent spread of *B. microti* babesiosis in the USA are presented in detail, which will make interesting and enlightening reading for all babesiologists. A range of interventions are described, and although their implementation has proved disappointing, the authors remain optimistic that by the centennial of the discovery of ‘Nantucket fever’, technological advances will have resolved many of the control and prevention problems. In the second review on *B. microti*, Goethert [8] describes its worldwide diversity, knowledge of which has evidently increased markedly over the decades since the parasite’s emergence as a human pathogen. The author argues that because many of the studies on *B. microti *were conducted before the availability of molecular analysis, an understanding of the ecology of *B. microti *has been hampered by confusion about parasite identity. They have now been taxonomically allocated to five distinct clades within the species complex, but problems with identity evidently persist in some recent studies.

Parasite diversity has also drawn the attention of researchers in the study of *B. divergens*-like pathogens since the occurrence of four human cases in the USA [9,10,11,12] and two in Europe [13,14]. In some of these reports, the infectious agent was initially identified as *B. divergens*, but subsequent analysis has established that they are all clearly distinct from this species and are currently described as *B. divergens*-like or have been given an abbreviation to indicate the location of the case. Thus, the causal agent of the first of these [9] occurred in Missouri and is described as *Babesia* sp. MO1. In this Special Issue, Bonsergent et al. [15] describe an isolate obtained from a case in France, which caused a usually mild infection, compared with classic *B. divergens* babesiosis. The subsequent molecular analysis determined that the parasite involved, which they name *Babesia *sp. FR1, belongs to the MO1 clade. This study demonstrates that variations in the severity of suspected *B. divergens *babesiosis [16] may be due to infections with *B. divergens*–like parasites rather than with the classic *B. divergens* of cattle. The reservoir host of *Babesia *sp. MO1 is believed to be the cotton-tail rabbit (*Sylvilagus floridanus*), and while the reservoir host of *Babesia *sp. FR1 is unknown, the European rabbit (*Oryctolagus cuniculus*) is implicated by its high abundance in the habitat where the infection is thought to have been contracted.

The list of zoonotic *Babesia *spp. is gradually lengthening, but it is difficult to determine the tick vector involved in the transmission of parasites known only as isolates from patients. The detection of parasite DNA in ticks is only indicative of vector status and absolute proof requires experimental demonstration of transmission under controlled conditions [17]. A review of the approaches and technologies to achieve such proof is presented by Bonnet and Nadal [18], who discuss the application of ticks to both naturally infected and experimental animals and also the increasing use of artificial tick-feeding systems. They conclude that systems for the experimental infection of ticks are vital tools for the determination of vector competence, enhancing our knowledge of pathogen ecology and of *Babesia *spp. life cycles, and consideration should be given to the standardisation of artificial-feeding protocols.

Although tick transmission is the primary means by which *Babesia *spp. infect humans, blood transfusions are an increasingly important source of infection, particularly of *B. microti* in the USA. Bloch et al. [19] review the history of transfusion-transmitted babesiosis, mainly in the USA, evaluate the evolution of surveillance, assay development, and screening policy in the USA, and suggest that the current American model for the prevention of transfusion babesiosis could form the basis for similar measures in other countries where the perception of transfusion transmission risk is currently low. One of the groups of patients that is particularly prone to haemolysis and require frequent blood transfusions are those suffering from haemoglobinopathies such as sickle cell anaemia and thalassaemia. Little is known about the course of babesiosis in such patients, but it has been accepted for many years that haemoglobinopathies afford some protection against malaria, and studies on *Babesia *spp. in this context, reviewed here by Beri et al. [20], suggest that such conditions also hinder intraerythrocytic growth of parasites. Possible mechanisms for the resistance of sickle cells to *Babesia *spp. are explored and suggestions are made for further studies to identify the possible ‘Achilles heel’ of both *Babesia* and *Plasmodium *spp. that could result in effective interventions.

The detection of *Babesia *parasites in stored blood by molecular methods is an essential component of screening procedures for blood transfusion and is also the most reliable approach for detection of parasites in clinical cases when parasitaemias are low, whereas microscopy in the hands of experienced laboratory staff is useful at higher parasitaemias. Meredith et al. [21] address the history, current status, and future prospects for laboratory diagnosis of *B. microti*, with particular emphasis on the application of modern technologies such as exploitation of the CRISPR–Cas system, which markedly increases the sensitivity of nucleic acid test systems. Serological testing for babesiosis has mainly relied on immunofluorescence techniques to detect surface antigens, and increased knowledge of the nature of these surface antigens is important. Delbecq [22] reviews the major surface antigens of *B. microti *and *B. divergens*, highlighting their role in both erythrocyte invasion and the immune response. He concludes that the increased knowledge of the major antigens will contribute to the development of vaccines, and of more sensitive serological assays and antigen capture assays that could be used to identify biomarkers for exposure, active infection, and protection. Other antigens, members of the rhoptry-associated protein-1 (pRAP-1) family, are the subject of a paper by Bastos et al. [23]. These proteins are secretory products of the apical complex in piroplasms, which plays an essential role in cell invasion by the parasite. Rhoptry proteins have not received the attention they should and the study described here suggests the involvement of pRAP-1 in parasite adhesion, attachment, and possibly evasion of the immune response. Antibodies in *B. microti*-infected humans recognise recombinant forms of the two proteins studied, suggesting that they could be candidates for both diagnostic assays and vaccines.

Efficacious drug treatment of patients is central to the management of babesiosis and a review of antimicrobial use in the past and present by Renard and Ben Mamoun [24] draws attention to the fact that the currently available drugs are limited and have been repurposed rather than developed specifically as antibabesials. Since they are associated with either significant side effects or the rapid emergence of drug resistance, it is clear that new therapeutic strategies are required. In vivo models for antibabesial evaluation using mice, hamsters, and gerbils have been available for some years but continuous culture in vitro has been restricted to *B. divergens*. The recent development of such a system for *B. duncani* is a major advance and, in combination with in vivo systems, is likely to result in *B. duncani* becoming the species of choice for the discovery of antimicrobials against all the zoonotic *Babesia* spp.

*Babesia microti* is the predominant zoonotic species and is also arguably the least susceptible to existing antimicrobials [25]. The identification of chemotherapeutic targets in these parasites thus becomes an important research priority. Florin-Christensen et al. [26] focus on species-specific proteases and have used bioinformatics to identify genes in the *B. microti* genome that code for these enzymes. They classify 89 proteases into five groups and report that comparisons between *B. microti *and *B. bovis *reveal differences between sensu lato and sensu stricto parasites, reflecting their distinct evolutionary histories, which is probably relevant to their susceptibilities to antibabesials [25]. In another paper on proteases [27] Šnebergerová et al. investigate aspartyl proteases in *B. microti*, particularly in relation to homologues of known function in other parasites, such as plasmepsins in *Plasmodium *spp. They suggest that analogies with plasmodial plasmepsins indicate piroplasmid aspartyl proteases as potentially important therapeutic targets.

We hope this Special Issue will motivate research scientists to further develop strategies for the prevention and control of babesiosis in the future. Improvements are required in diagnosis, the rigorous typing and identification of *Babesia* parasites, prevention of transfusion transmission, and the discovery of novel antibabesial drugs. The development of safe and effective vaccines for use in humans remains an unrealised goal and is an important research priority.

The researchers who have participated in this Special Issue remind us that zoonotic babesiosis is a complex emerging disease, in which ticks and domestic and wild animals have crucial roles so that environmental factors, particularly in a climate change context, must be taken into account. In the coming years, multidisciplinary collaboration between research groups, the use of digital tools for analysing and sharing essential data about current and new species, the involvement of health authorities in the implementation of surveillance systems, and the development of specific funding strategies for emerging infections such as babesiosis, will be decisive in achieving the necessary goals. Finally, it is imperative to inform and collaborate with veterinary scientists, community health care workers, and the general population in order to determine and reduce the risk of zoonotic babesiosis.
